# Navigating the syndemic: a socio-psychological approach to food security and responsible consumption in low-income populations

**DOI:** 10.3389/fnut.2026.1793312

**Published:** 2026-06-05

**Authors:** Nahui Samanta Nájera-Segura, Adrián Martínez-Vargas, Beatriz Chávez-Ceja, María del Carmen Avendaño-Rito, Eduardo Cruz-Cruz, Paola Miriam Arango-Ramírez, Hector Alejandro Cabrera-Fuentes, Sandra Nelly Leyva-Hernández

**Affiliations:** 1 Instituto Tecnológico del Valle de Etla, Tecnológico Nacional de México, Oaxaca, Mexico; 2 Centro de Investigación Facultad de Medicina UNAM-UABJO, Facultad de Medicina y Cirugía, Universidad Autónoma Benito Juárez de Oaxaca, Oaxaca, Mexico; 3 Unidad Coronaria y Urgencias Cardiovasculares, Instituto Nacional de Cardiología Ignacio Chávez, Ciudad de México, Mexico; 4 División de Estudios de Posgrado e Investigación, Instituto Tecnológico de Tijuana, Tecnológico Nacional de México, Tijuana, Mexico; 5 Hospital General de Zona No.1 Dr. Demetrio Mayoral Pardo, Instituto Mexicano del Seguro Social (IMSS), Oaxaca, Mexico; 6 R&D Group, Vice Presidency for Scientific Research and Innovation, Imam Abdulrahman Bin Faisal University, Dammam, Saudi Arabia

**Keywords:** food security, sustainable food systems, Health Belief Model (HBM), Theory of Planned Behavior (TPB), socially responsible consumption, short value chains, cognitive load, food insecurity

## Abstract

Global food security is currently a priority, especially amid a “grim regression” that is undermining efforts to eradicate malnutrition by 2030. This perspective argues that the current crisis is not merely a supply chain disruption but a syndemic convergence where biological threats and economic instability interact to erode consumer agency. We posit that vulnerable populations are forced into a “lethal calculation,” prioritizing immediate caloric survival over food security and long-term health, thereby becoming trapped in cycles of ultra-processed food consumption. To address this, we challenge the limitations of purely rational economic models and propose an integrated psychological framework that combines the Theory of Planned Behavior (TPB) and the Health Belief Model (HBM). This integration accounts for the specific “internal determinants” and “perceived threats” that drive decision-making in high-stress, low-income environments to achieve food system sustainability. Unlike previous studies that primarily apply the TPB and HBM to predict individual choices, this perspective uses their integration to propose a structural reorientation of food systems. We introduce the concept of “reverse-designed food systems” as a conceptual bridge that aligns system architecture with the cognitive and behavioral constraints of consumers in high-stress environments. This approach shifts the focus from production-led efficiency to a consumer-centric structural design, where the system infrastructure assumes the cognitive load traditionally borne by individuals living in poverty.

## 1 Introduction

In an era defined by overlapping global crises, the challenge of nutrition has increasingly extended beyond the traditional domains of agriculture and economics (1). Rather than a series of isolated supply disruptions, contemporary food insecurity is increasingly conceptualized as a syndemic, in which biological, social, and economic factors may interact synergistically to disproportionately affect vulnerable populations (2). This perspective suggests that unsafe and nutritionally poor consumption in low-income can be understood as the outcome of constrained decision-making under chronic stress, where immediate caloric needs frequently override longer-term health considerations. Prevailing food security and sustainable consumption frameworks emphasize availability, affordability, and rational choice, yet offer limited insight into why unsafe food practices persist even when healthier options are nominally present (3, 4). A more comprehensive approach may be needed, one that goes beyond the traditional framework of availability, access, utilization, and stability. Food security can also be understood from the perspectives of agency and sustainability (5), although this requires a behavioral understanding of food consumption and its underlying reasons. Despite increasing attention to food system sustainability, many frameworks tend to assume that consumers behave as rational economic actors capable of consistently making optimal food choices (6). However, behavioral science suggests that decision-making under conditions of poverty is often shaped by cognitive overload, stress, and limited agency (7, 8). In such contexts, food choices can be understood not merely as economic transactions but as adaptive responses to structural constraints. Consequently, understanding food security may require integrating insights from behavioral and health psychology into food system analysis.

To address this gap, this article argues that a comprehensive approach may be needed to analyze responsible consumption in vulnerable contexts, taking into account health and psychological variables; therefore, it may be necessary to incorporate diverse theoretical frameworks, such as the Theory of Planned Behavior (TPB) and the Health Belief Model (HBM). This integration may help to better capture how perceived health threats, reduced agency, and constrained behavioral control shape food security decisions in syndemic contexts (9). Building on this integration, a reverse-designed food system is proposed as a conceptual synthesis that aims to reorient food system design outward from consumer cognition rather than inward from production. This approach aims to extend existing short value chain and food environment literature by conceptualizing local food systems as trusted social infrastructures that reduce cognitive load and facilitate safer choices. From a policy perspective, this framing suggests that system-level design, rather than individual responsibility, may better support food security and sustainable consumption in low-income settings. The theoretical contribution of this work lies in employing the integrated TPB-HBM framework not merely to analyze purchasing intent, but also as a blueprint for the reverse design of food systems. While the synthesis of these models is well established in health psychology, their application to the configuration of trusted social infrastructures for vulnerable populations remains underutilized. We position “reverse-design” as a strategy to externalize perceived behavioral control, enabling systems such as Short Value Chains (SVCs) to automate healthy choices and reduce decision fatigue. Consequently, the proposed framework sits at the intersection of behavioral science and food system governance, offering a structural alternative to traditional models that overlook the psychological realities of the food syndemic.

## 2 The grim reality of a global regression

The contemporary landscape of global food security is currently undergoing a grim regression. This is not a subtle shift but a phenomenon that systematically undermines the Sustainable Development Goals established to eradicate hunger and malnutrition by the end of this decade. According to the critical findings presented, approximately 828 million people were affected by hunger in 2021 alone, representing a staggering increase of 150 million individuals since the onset of the COVID-19 pandemic (10). This crisis is far from a transient or isolated disruption; rather, it has evolved into a complex syndemic in which the virus’s biological threat interacts synergistically with long-standing social and economic instability. Crucially, a syndemic is not merely the co-occurrence of problems, but their destructive mutual enhancement (11). In this context, the biological threat of the virus and the economic threat of poverty conspire to erode consumer agency (12). This syndemic convergence may predispose consumers to a trade-off between the immediate survival need for calories, which overrides the longer-term imperative for food security (2).

The profound dysfunction of the current global food system is increasingly characterized by a widening chasm between caloric availability and nutritional affordability (13). While geopolitical conflicts, the escalating climate emergency, and systemic supply chain disruptions have significantly driven up the cost of healthy diets, national governments continue to provide support that is overwhelmingly directed toward cereals and staples rather than nutrient-dense foods (14). Thus, not only is it necessary to mitigate food insecurity, but it is also extremely urgent to manage a global obesity crisis, driven by the high availability of cheap, high-calorie foods (10). Consequently, there is an imperative need to promote sustainable diets, which, as defined by their low environmental footprint and capacity to protect biodiversity, represent a fundamental pillar for the future of public health. However, shifting toward such models requires more than structural change; it necessitates a deep understanding of the psychological determinants that guide consumer behavior in high-stress environments.

## 3 Food security and Socially Responsible Consumption

The concept of food security has transitioned from a narrow preoccupation with aggregate food supply to a nuanced, multidimensional framework encompassing availability, access, utilization, and stability (1). This evolution acknowledges that food must not only be available in the market but also be safe (intrinsically linked to food safety), culturally appropriate, and nutritionally adequate to meet a population’s dietary needs. Household food insecurity is consistently linked to chronic undernutrition, increased susceptibility to infectious diseases, and significant developmental issues in children, including impaired academic performance and persistent behavioral struggles (2). Furthermore, a new emphasis on nutrition aims to align agricultural production more closely with human health needs, seeking to rectify the historical disconnect between what we grow and what we need to survive and thrive.

To counteract these deep-seated inequities, researchers have turned their attention to local food systems, such as SVC models (15–17). SVC models, such as producing prescriptions, are identified as critical strategies for enhancing fruit and vegetable intake in marginalized communities (15). However, even these local solutions face significant barriers to engagement, ranging from a lack of program awareness to logistical challenges such as transportation and cultural incongruencies that prevent vulnerable families from fully utilizing these resources.

Despite these theoretical advancements, the reality on the ground remains alarming. The global prevalence of adult obesity continues to trend upward, driven by economic incentives that make obesity-conducive foods far more accessible than healthy options (10). In this context, Socially Responsible Consumption emerges as an ethical and health-related imperative. This form of consumption is inextricably linked to the adoption of sustainable diets; nevertheless, this transition presents a complex behavioral challenge (18). Sustainable food choices are deeply connected to psychological well-being and require a comprehensive understanding of the internal determinants, such as self-identity and personal values, and the external determinants, such as social support and information provision, that guide modern consumers. Food waste in low-income settings is not merely a logistical failure but an emergent behavioral outcome of chronic stress and constrained decision-making. Under economic insecurity, households often adopt risk-averse purchasing strategies, such as buying larger quantities of cheaper foods to ensure short-term caloric sufficiency. However, this coping mechanism paradoxically increases household food waste through accelerated spoilage, inadequate storage, and precautionary discard driven by mistrust in food security. Cognitive overload and time scarcity further impair meal planning and food utilization, reinforcing a cycle in which both nutritional quality and resource efficiency are compromised. In this context, SVCs function as waste-preventive infrastructures rather than mere distribution channels. By improving freshness, transparency, and social trust between producers and consumers, SVCs reduce spoilage, limit mistrust-driven food rejection, and enable smaller, more frequent purchases that align with household capacity. From a sustainability perspective, this behavioral reduction in household-level food waste represents a critical, yet often overlooked, circular-economy gain achieved through system design rather than individual behavioral correction. To facilitate this shift, Table 1 outlines how behavioral determinants can be translated into specific reverse-design principles for food systems.

**Table 1:** Operationalizing the reverse-designed food system.

Behavioral determinant	Reverse-design principle	Practical food system action	Policy relevance
Perceived behavioral control	Reduce decision complexity	Neighborhood SVCs with curated offerings	Municipal food planning
Subjective norms	Embed food access in social contexts	Community markets and cooperatives	Social cohesion policies
Perceived severity	Make food risk visible	Traceability and vendor transparency	Food safety regulation
Perceived benefits	Align health and convenience	Fresh, culturally relevant foods	Nutrition-sensitive subsidies
Cognitive load	Automate responsible choice	Default healthy food bundles	Public procurement programs
Self-efficacy	Build trust-based systems	Stable producer–consumer relationships	Local capacity-building

## 4 Behavioral foundations of food security: integrating the HBM and the TPB

To understand why individuals in vulnerable contexts choose certain foods over others, we must turn to robust psychological models. The TPB has served as the dominant framework for predicting sustainable consumption (19, 20). This model posits that human behavior is driven by the interaction of three constructs: attitudes toward the behavior, subjective norms reflecting social pressure, and perceived behavioral control, which accounts for the perceived ease or difficulty of performing the action (21). A meta-analysis confirms that these variables are significantly associated with intention, though the strength of these relationships can vary depending on the nature of the food choice and the consumer’s age (22).

The application of TPB becomes even more complex when viewed through a cross-cultural lens (23). In developing countries, subjective norms and perceived behavioral control play a significantly more critical role in shaping purchase intentions compared to Western cultures (24). This suggests that, in the Global South, individual choices are heavily influenced by social pressure and the practical availability of products. Furthermore, while Western populations often integrate personal norms and self-identity as green consumers, individuals in non-Western cultures are more frequently driven by collective environmental concerns and health consciousness (25). These cultural nuances demonstrate that a one-size-fits-all approach to promoting sustainable diets is unlikely to succeed if it fails to account for the specific social pressures of the target community.

To address the limitations of a purely rational model like the TPB, contemporary research strongly advocates for an integrated approach with the HBM. While the TPB excels at explaining volitional-oriented behavior, the HBM accounts for internal health-related motivations and perceptions of risk (26). In a study of young Pakistani households, combining these models provides a superior explanation for intentions to prevent food waste (27). By making the perceived threats of food insecurity and the perceived benefits of conservation more salient, practitioners can foster a stronger sense of urgency. Moreover, this integration enables the inclusion of personality traits, such as food neophilia and neophobia (28). Understanding whether consumers are inherently reluctant to try new, sustainable foods or are motivated by the novelty of healthy alternatives enables more targeted, effective marketing strategies to overcome psychological resistance.

While integrating TPB and HBM provides a comprehensive lens for understanding food choices, it is critical to acknowledge their inherent limitations when applied to populations facing chronic scarcity and syndemic stress. Most behavioral models, including these two, are historically rooted in a “rational actor” paradigm, which assumes a baseline level of individual agency that is often absent in low-income settings (6).

The primary limitation of the TPB in vulnerable contexts is its reliance on perceived behavioral control as a predictor of intention. In a syndemic environment—where biological threats and economic instability converge—the “control” an individual perceives is frequently a reflection of overwhelming external barriers rather than internal capability (29). For consumers trapped in a “lethal calculation” between immediate caloric survival and long-term health, the TPB’s focus on individual attitudes and intentions may overlook the fact that structural constraints (e.g., lack of refrigeration, proximity to food deserts) render personal intentions secondary to systemic necessity (22). Consequently, the model risks overestimating individual agency while underestimating the “syndemic erosion” of choice (30).

The HBM focuses on perceived severity and susceptibility to health threats (26). However, in contexts of extreme poverty, the salience of long-term health risks (such as obesity or metabolic disease) is often diminished by the urgency of immediate hunger—a psychological phenomenon known as temporal discounting (31). When survival is the primary driver, the “perceived benefits” of a sustainable diet (central to the HBM) cannot compete with the immediate benefit of inexpensive, calorie-dense ultra-processed foods (32). Thus, the HBM’s effectiveness is limited because it assumes that health-related risk perception is the primary motivator, failing to account for how cognitive overload and high-stress environments shift priorities toward short-term caloric security (7, 32).

Both models struggle to explain the significant “intention–behavior gap” observed in low-income populations (33). Even when consumers possess positive attitudes and high risk-awareness, the mental energy required to navigate complex, unsafe, or unaffordable food environments creates a cognitive load that paralyzes decision-making (8, 30). This is why traditional interventions focusing on education (targeting attitudes) or fear-based messaging (targeting perceived severity) often yield poor results in these settings (34).

## 5 Proposed framework

By recognizing these limitations, it becomes clear that the solution lies not in demanding more “rationality” or “responsibility” from consumers, but in redesigning the system itself. The proposed framework, therefore, uses the TPB and HBM not as predictors of individual success but as diagnostic tools to identify where the system must intervene to “externalize” the control and “automate” the choices that the consumer’s current environment makes impossibly difficult.

The conceptual novelty of reverse-designed food systems transcends conventional consumer-centered food environments and behavioral architecture by proposing a constitutive, rather than merely elective, structural change. While traditional behavioral interventions, such as TPB (Traditional Behavioral Policies), tend to focus on modifying individual decisions within existing production frameworks (21), the reverse-design approach posits that the food system infrastructure must be designed outward, starting from the specific cognitive limitations and syndemic erosion of the capacity for action experienced by vulnerable populations. This proposal builds on the work of Guerrero Lara et al. (35), who argue that innovation in the agri-food system is based on safeguarding agrobiodiversity, cultural heritage, and the social inclusion of marginalized and vulnerable populations, as well as O’Connor et al. (17), who indicate that the experiences of local producers are key to the transition to sustainable food systems.

In this context, the food environment is not simply a space of availability or affordability; it is redefined as a trusted social infrastructure specifically designed to externalize perceived behavioral control. This infrastructure promotes community participation in identifying needs, developing products, and connecting with other organizations to increase visibility, thereby empowering the community and improving the sustainability of local initiatives (36). This approach differs from standard system-level interventions by prioritizing cognitive load reduction as the main organizing principle of the supply chain. In doing so, it transforms SVCs from simple logistical channels into mechanisms geared toward responsible consumption (37), thus mitigating the lethal calculation imposed by the high-stress environments of the food syndemic.

**Figure 1:**
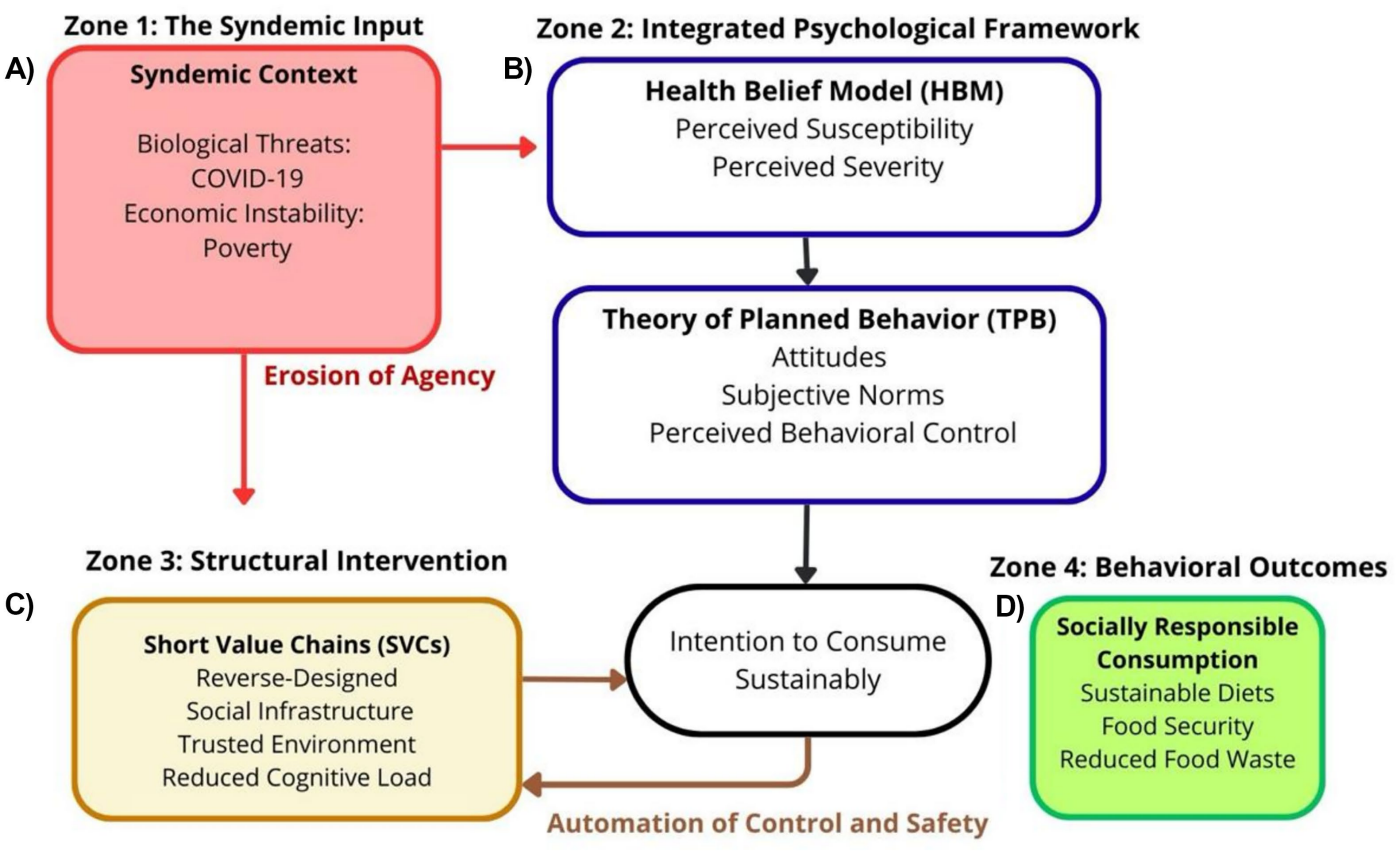
The reverse-designed food system framework. This conceptual diagram illustrates the proposed pathway for mitigating food insecurity in syndemic contexts. **(A)** Syndemic input: Biological and economic threats converge to erode consumer agency, forcing a “lethal calculation” between survival and safety. **(B)** Integrated psychological engine: The framework synthesizes the TPB and the HBM. Recent empirical evidence supports this integration for predicting health behavior intentions (9) and food waste prevention (27), specifically accounting for personality traits such as neophobia (28). **(C)** Structural intervention: SVCs are positioned as “reverse-designed” social infrastructures. By functioning as trusted environments (17), SVCs reduce cognitive load and automate perceived behavioral control, a critical predictor of consumption in non-Western contexts (24). This leads to **(D)** SociallyResponsible Consumption, characterized by reduced waste and improved nutritional security.

Figure 1 presents the core of the proposed behavioral–structural framework, illustrating the transition from vulnerability to sustainable food security through a reverse-design approach. The framework begins by acknowledging that food insecurity in low-income populations is a syndemic destructive convergence of biological threats (such as COVID-19) and chronic economic instability (1, 5). This interaction may predispose consumers to a “lethal calculation,” in which the immediate need for caloric intake to survive overrides long-term health and food safety considerations (2, 11).

This context systematically erodes consumer agency, making traditional “rational choice” models insufficient for explaining behavior. To capture the internal drivers of decision-making under stress, the framework integrates the Theory of Planned Behavior (TPB) and the Health Belief Model (HBM). By combining these models, the framework can address both volitional behaviors and risk-related motivations, even accounting for personality traits like food neophobia (reluctance to try new foods) (9, 27).

The “integrated engine” informs the design of Reverse-Designed Food Systems, which are configured from the consumer’s cognitive constraints outward rather than from production inward. SVCs are repositioned not just as logistical channels, but as trusted social environments, understood as local markets where there is an interpersonal relationship between the producer and the consumer. By leveraging localized markets and curated offerings, the system “externalizes” perceived behavioral control, which can be operationalized as the degree to which the system itself makes decisions by providing predefined choices rather than allowing the consumer to make free selections. This essentially “automates” responsible choices, reducing the mental effort and decision time required to compare potential purchase options (cognitive load) for vulnerable consumers navigating complex or unsafe food environments (17, 24).

The ultimate goal of this pathway is Socially Responsible Consumption, characterized by improved nutritional security and reduced food waste. In this framework, reducing waste is not merely a matter of individual discipline but a behavioral outcome of a system that provides freshness, transparency, and trust, thereby eliminating the need for risk-averse, “precautionary” purchasing strategies that lead to spoilage.

In summary, Figure 1 proposes that while syndemic pressures erode individual agency, a reverse-designed system can restore it by aligning the food system architecture with consumers’ psychological realities. In syndemic contexts, high cognitive load reduces perceived behavioral control, leading to decisions to consume low-cost, ultra-processed foods. SVCs designed according to the principles of “reverse design” increase consumer self-efficacy by reducing uncertainty about food safety. To further clarify how the behavioral components of the TPB and the HBM shape food decision-making, Table 2 summarizes the key psychological determinants influencing food choices in vulnerable populations.

**Table 2:** Psychological determinants influencing food choices in vulnerable populations: an integration of the TPB and the HBM*.

Determinant	TPB component	HBM component	Relevance for food security
Attitudes	Attitudes toward behavior	Perceived benefits	Influences willingness to adopt healthier diets
Social influence	Subjective norms	–	Community norms affect food choices
Behavioral capacity	Perceived behavioral control	Self-efficacy	Determines the ability to access healthy food
Risk perception	–	Perceived severity and susceptibility	Drives motivation to avoid unsafe foods

* The integration of Theory of Planned Behavior (TPB) and Health Belief Model (HBM) highlights how both social influences and perceived health risks jointly shape food decisions in vulnerable populations.

## 6 Conclusions and policy implications

The integration of the TPB and the HBM provides a vital psychological foundation for addressing the multifaceted global food security crisis. The variables within this theoretical framework may help address the qualitative aspects of food insecurity (38). The pleasure derived from eating falls under the TPB’s attitudes variable, while norms, family relationships, and culinary traditions are under subjective norms. Nutritional and health education are reflected in the Human Behavior Model. The transition toward sustainable and socially responsible habits may require a strategic focus on the cognitive determinants of choice that go beyond simple economic incentives. As evidenced by the systematic reviews, targeting internal constructs such as self-efficacy and autonomous motivation appears essential for bridging the gap between a consumer’s intention to eat healthily and their actual behavior (18, 39). For populations in vulnerable contexts, these psychological facilitators are often suppressed by overwhelming external barriers, suggesting that interventions may need to address both the mind and the environment. Furthermore, eating disorders have been associated with food insecurity in marginalized populations, particularly among adolescents (40). Therefore, the analysis of behavioral aspects may benefit from further development, and this perspective may serve as a basis for a more holistic behavioral analysis of food insecurity.

It is recommended that future studies address the theoretical implications of the proposed model. Specifically, it is suggested that future research empirically analyze whether reducing cognitive load through simplified systems (such as pre-selected food packages) can increase perceived behavioral control by decreasing the mental effort required to assess food safety in contexts of chronic stress. Furthermore, it is proposed that studies analyze how social infrastructures of trust can influence the relationship between subjective norms and consumption intentions, transforming social pressure into a facilitator of self-efficacy. Finally, it is proposed that studies empirically analyze how externalizing behavioral control can reduce the gap between intention and behavior, enabling responsible consumption even when individual agency is diminished by syndemic factors.

From a policy perspective, these findings demand a paradigm shift. The transition toward reverse-designed food systems requires a departure from traditional, production-centric policies toward interventions that actively “externalize” the psychological and logistical burdens currently placed on vulnerable consumers.

Current policies often disproportionately subsidize calorie-dense staples like cereals, which inadvertently fuel the obesity crisis in low-income settings. Governments should redirect these funds toward nutrition-sensitive subsidies that specifically target fresh, culturally relevant foods available in local markets. A practical example is the implementation of produce prescription programs and digital vouchers redeemable only at neighborhood SVCs. This strategy not only makes healthy food affordable but also aligns with the consumer’s “perceived benefits,” fostering long-term dietary shifts.

To address cognitive load and decision fatigue, public policy must focus on “automating” responsible choices through system-level design. Public procurement programs—such as school lunch initiatives and social assistance packages—should adopt default healthy food bundles. By providing pre-curated selections of fresh produce and minimally processed foods, the system reduces the “decision-making waste” and the “lethal calculation” forced by stressful environments, making the healthy choice the easiest and most accessible option by default.

Rather than viewing local markets solely as logistical nodes, policy must treat them as tools for social cohesion that leverage community trust. Municipal food planning should prioritize the creation of neighborhood-based SVCs and cooperatives that reduce physical distance and logistical barriers. By embedding food access within familiar social environments, these markets serve as “trusted social infrastructures” where subjective norms and social transparency validate food safety and quality, thereby eliminating the need for individuals to constantly verify food safety in conditions of uncertainty.

Policy must move toward decentralized food systems that empower both producers and consumers through stable relationships. Regulations should favor traceability and vendor transparency to increase the “perceived severity” of food risks and the “perceived benefits” of safe local sourcing. This includes funding for local capacity-building and creating convenient, “friendly” market environments that reflect the community’s specific culture, thereby increasing consumer self-efficacy. In summary, by shifting the policy level from individual responsibility to systemic reverse-design, governments can mitigate the erosion of agency caused by the food syndemic and foster a more equitable and resilient food landscape.

This illustrates how integrating two frameworks may contribute to a more comprehensive understanding of food systems and to the promotion of public policy strategies that aim to mitigate food insecurity by emphasizing consumer motivations. Future research could empirically test the proposed framework across diverse socioeconomic settings and evaluate how reverse-designed food systems influence consumer decision-making, dietary quality, and food security outcomes in vulnerable populations. Such investigations may help translate the conceptual insights of this perspective into actionable strategies for sustainable and equitable food systems.

## Data Availability

The original contributions presented in the study are included in the article/supplementary material, further inquiries can be directed to the corresponding authors.
